# Protective Effect of Hawthorn Fruit Extract against High Fructose-Induced Oxidative Stress and Endoplasmic Reticulum Stress in Pancreatic β-Cells

**DOI:** 10.3390/foods12061130

**Published:** 2023-03-08

**Authors:** Hsiu-Man Lien, Hsin-Tang Lin, Shiau-Huei Huang, Yìng-Ru Chen, Chao-Lu Huang, Chia-Chang Chen, Charng-Cherng Chyau

**Affiliations:** 1 Research Institute of Biotechnology, Hungkuang University, Shalu District, Taichung 43302, Taiwan; 2 SYi Biotek, 2F, No. 26, Keyuan Rd., Xitun District, Taichung 40763, Taiwan; 3 Graduate Institute of Food Safety, National Chung Hsing University, 145, Xingda Road, Taichung 40227, Taiwan

**Keywords:** hawthorn fruit, *Crataegus pinnatifida*, polyphenol-enriched extract, pancreatic β-cell, hyperglycemia, endoplasmic reticulum (ER) stress, gene expression

## Abstract

Hyperglycemia has deleterious effects on pancreatic β-cells, causing dysfunction and insulin resistance that lead to diabetes mellitus (DM). The possible causes of injury can be caused by glucose- or fructose-induced oxidative and endoplasmic reticulum (ER) stress. Hawthorn (*Crataegus pinnatifida*) fruit has been widely used as a hypolipidemic agent in traditional herbal medicine. The study aimed to investigate whether high fructose-induced pancreatic β-cell dysfunction could be reversed through amelioration of ER stress by the treatment of polyphenol-enriched extract (PEHE) from hawthorn fruit. The extract was partitioned using ethyl acetate as a solvent from crude water extract (WE) of hawthorn fruits, followed by column fractionation. The results showed that the contents of total polyphenols, flavonoids and triterpenoids in PEHE could be enhanced by 2.2-, 7.7- and 1.1-fold, respectively, in comparison to the original obtained WE from hawthorn fruit. In ER stress studies, a sharp increase in the inhibitory activity on the gene expression levels of GRP79, ATF6, IRE1α and CHOP involved in ER stress was evident when dosages of PEHE at 50–100 μg/mL were used against high-fructose (150 mM)-treated cells. HPLC–MS/MS analysis showed that polyphenols and flavonoids collectively accounted for 87.03% of the total content of PEHE.

## 1. Introduction

The dried fruit of Hawthorn (*Crataegus pinnatifida*), a member of the Rosaceae family widely distributed in North America, Europe and Asia [1], is a famous fruit valued for its use with digestion problems. It has been used widely for preventing and treating cardiovascular diseases in China, Europe and the USA [1,2]. Ethnopharmacological studies reported that hawthorn fruit has been used for its anti-inflammatory, anticancer, anti-cardiovascular disease and digestion-enhancing properties, as well as for dissipating blood stasis [2,3,4]. There is also growing evidence indicating an enhancing effect of hawthorn fruit on plasma insulin secretion levels in streptozotocin (STZ)-induced type II diabetes mellitus (DM) rats [5]. 

The phytochemical composition of hawthorn fruit is rich in polyphenols, including flavonoids, phenolic acids and procyanidins (PC) [6,7]. In addition, hawthorn fruits contain significant amounts of triterpenoids and lignans [8,9]. Compositional investigations of three varieties of *C. pinnatifida* fruits reported high amounts of total phenolic content in the results of 31.4 to 104.6 mg gallic acid equivalents per g in the dry weight of fruits [10]. In a quantitative analysis of phenolic compounds on *C. pinnatifida* fruits, chlorogenic acid, hyperoside (quercetin-3-*O*-galactoside), isoquercitrin (querce-tin-3-*O*-glucoside), epicatechin (EC) and epicatechin-(4β → 8)-epicatechin (PC-B2) were indicated as the major compounds [11]. Moreover, the two most abundant components in the fruits were indicated from EC and PC-B2, with contents of 348 and 374 mg/100 g, respectively [12]. EC has been found to elevate insulin sensitivity as well as to lower insulin resistance [13]. PC-B2, composed of two molecules of EC linked by a C4-C8 bond, has been reported to prevent reactive oxygen species (ROS) generation and inhibit inflammation [14], and to protect cells from hyperglycemia-induced dysfunction [15]. Therefore, preparing enriched polyphenols extract, especially EC and PC-B2 from hawthorn fruit to exert the pancreatic β-cell function, could be a useful therapeutic strategy for hyperglycemia-induced injury. 

There are several factors involved in inducing diabetes progression, including hyperglycemia- and hyperlipidemia-induced oxidative stress and inflammation [16,17]. In the physiological environment, pancreatic beta cells are extremely sensitive to oxidative stress due to their low intracellular antioxidant capacity [18,19]; in particular, the expressions of key antioxidant enzymes, catalase and glutathione peroxidase (GPx) are much lower in β-cells than in α-cells [19]. These pathogenic factors are important in leading to apoptosis and a decrease in beta cell mass [20]. Hawthorn fruits have been used to treat symptoms such as cardiovascular disease (CVD) [21], gastrointestinal motility disorder [22] and inflammation [23]. Furthermore, a broad spectrum of pharmacological actions on the neuroprotection, antibacterial, antiviral, anti-diabetes, anti-aging, anti-obesity and other actions have been reported in hawthorn fruits [24], but very little is known about the protective effects on beta cells of hawthorn fruit against high-fructose-induced endoplasmic reticulum (ER) stress and cell apoptosis. Based on previous reports indicated that the antioxidants can be act as ER stress inhibitors in DM, the aim of this study was to investigate the potential inhibition efficacy on ER stress of PEHE.

## 2. Materials and Methods

### 2.1. Materials and Chemicals

Bright red and ripe hawthorn (*Crataegus pinnatifida*) fruits were collected in December 2021 from a local farm in Taichung, Taiwan. The air-dried samples were identified using micro- and macroscopic description in the Taiwan Herbal Pharmacopeia [25], as well as in comparisons with herbarium specimens kept in the Research Center for Biodiversity, Academia Sinica, Taipei, Taiwan. A voucher specimen (No. 110-1) was deposited in the Research Institute of Biotechnology, Hungkuang University, Taichung, Taiwan.

(−)-Epicatechin, hyperoside (Quercein-3-*O*-galactoside), procyanidin B2 and isoquercitrin (Quercein-3-*O*-glucoside) were purchased from ChemFaces (Wuhan, China). Antibodies against β-Actin (tcba13655), GRP78 (tcea19663) and CHOP (tcba1658) were purchased from Taiclone (Taipei, Taiwan). HPLC-grade methanol, acetonitrile and ultrapure water, trypan blue, 3-(4,5-dimethyl thiazol-2-yl)-2,5-diphenyltetrazolium bromide (MTT), butylated hydroxytoluene (BHT), Trolox, *p*-nitrophenyl-α-D-glucopyranoside (*p*NPG) and 1,1-diphenyl-2-picrylhydrazyl radical (DPPH) andα-glucosidase (from *Saccharomyces cerevisiae*) were purchased from Sigma-Aldrich Chemical Co., St. Louis, MO, USA. Fetal Bovine Serum (FBS), L-glutamine solution (100 mM) and penicillin–streptomycin (5000 units/mL and 5 mg/mL streptomycin) were purchased from Biological Industries (Beit Haemek, Israel). Dulbecco’s Modified Eagle Medium (DMEM) and trypsin-EDTA solution were provided by Hyclone (Logan, UT, USA). The protein assay kit was a product of Bio-Rad (Hercules, CA, USA). The ESI-L low concentration tuning mix (G1969-85000) was purchased from Agilent Technologies (Santa Clara, CA, USA). 

### 2.2. Preparation of Hawthorn Fruit Extract 

After removing the seeds, the oven-dried and grounded hawthorn fruit powders (10.0 g) were extracted with 200 mL of distilled water under stirring for 1 h at 90 °C. The water extracts were centrifuged at 6000 rpm for 5 min at 4 °C. Collected supernatants were then evaporated and dried in a rotary evaporator under vacuum at 40 °C to afford 2.49 ± 0.65 g of hawthorn fruit extracts. The concentrated water extract (WE) was suspended in water (50 mL), and was partitioned with ethyl acetate (50 mL × 3 times). The obtained ethyl acetate extracts were combined and dried under vacuum at 40 °C to provide the ethyl acetate (EA) fraction. The EA fraction was dissolved in 70% methanol and was subjected to a Sephadex^®^ LH–20 column (id × L = 1.5 × 30 cm) for washing with distilled water and eluting with 70% of methanol at a flow rate of 1 mL/min in a total volume of three bed volumes (BV), respectively. The 70% methanol fraction was dried with rotatory evaporation under vacuum at 40 °C to afford 0.31 ± 0.02 g (designated as PEHE). 

### 2.3. HPLC and HPLC/ESI–MS/MS Analysis of PEHE 

The analysis of major polyphenols and flavonoids of PEHE was determined in accordance with a previous report [11], using a HPLC/electrospray ionization (ESI) triple quadruple mass spectrometer. In brief, a Kinetex^®^ EVO C18 column (100 × 2.1 mm, 2.6 µm) with a Security-Guard Ultra C18 guard column (2.1 mm × 2.0 mm, sub-2 µm, Phenomenex, Inc., Torrance, CA, USA) using an HPLC system consisted of a photodiode-array detector (DAD) that was applied to the separation of components in PEHE. The gradient elution system using two solvents was as follows: Solvent A (formic acid/water, 0.1:99.9, *v*/*v*) and Solvent B (formic acid/acetonitrile, 0.1:99.9, *v*/*v*). The flow rate of the mobile phase was 0.3 mL/min, and the column temperature was 35 °C. The gradient program was conducted as follows: 0–3 min (2% B), 3–23 min (2–35% B), 23–30 min (35–95% B), 30–40 min (95% B) and 40–45 min (95–2% B). The absorption spectra of eluted compounds were recorded using the in-line DAD over a wavelength range of 210–600 nm, and the peaks were monitored at 254, 280 and 320 nm, respectively. The compounds were eluted and separated, and further identified with a triple quadruple mass spectrometer (Agilent 6420, Santa Clara, CA, USA). The mass spectrometer was operated in both positive and negative ionization modes with a potential of ±3500 V, respectively. The injection volume was 10 μL using an autosampler. The drying gas was nitrogen (9 L/min), and the pressure of the nebulizing gas was 35 psi. The drying gas temperature was 325 °C. The fragmentor voltage was 125 V, and the in-source collision-induced dissociation (CID) voltage was 15 V. The collision gas was nitrogen. The ions produced by nitrogen collision were detected in the range of 100–1200 amu at a scan time of 200 ms/cycle. The MS instrument was externally calibrated with ESI-L Low Concentration Tuning Mix. The identification of separated compounds was carried out by comparing the retention times, UV-Vis spectra and mass spectra provided by ESI-MS and ESI-MS/MS with those of authentic standards when available, or the data reported from literature.

### 2.4. Determination of Antioxidants

#### 2.4.1. Total Polyphenolic Content (TPC)

The previous method [26] was followed for the determination of total phenolics. Briefly, WE or PEHE extract was dissolved into methanol at a concentration of 1 mg/mL. The sample solution (0.1 mL) was added into 2 mL of Na_2_CO_3_ (2%). After two min, the Folin–Ciocalteau reagent mixture (0.1 mL, 50%) was added and left to stand for 30 min. The absorbance was detected at 750 nm using a microplate reader (ELx800, BioTek Instruments Inc., Winooski, VT, USA). A calibration curve was similarly established using authentic gallic acid. The amount of phenolics was expressed as mg of gallic acid equivalent per g of dried weight of hawthorn fruit (mg GAE/g DW). 

#### 2.4.2. Total Flavonoid Content (TFC)

The assay for total flavonoids was carried out according to the method described [27]. In brief, 250 μL of sample solution (5 mg/mL) and deionized water (1.25 mL) were added into NaNO_2_ solution (75 μL, 5% *w*/*v*) and mixed well, standing for 6 min. In Then, 150 μL of AlCl_3_·6H_2_O (10% *w*/*v*) solution was added and left to stand for 5 min. Next, 0.5 mL of 1 M NaOH and 2.0 mL of deionized water were added and mixed well. The absorbance of the solution was detected at 510 nm. A calibration curve was established using authentic quercetin. The amount of flavonoids was expressed as mg quercetin equivalent per g of dried weight of hawthorn fruit (mg QE/g DW). 

#### 2.4.3. Total Triterpenoid Content (TTC)

The content of the total triterpenoids was determined with the vanillin/glacial acetic acid method [28]. The 100 μL of methanolic WE or PEHE solution (1 mg/mL) was added into 150 µL of 5% vanillin/glacial acetic acid (*w*/*v*), followed by 500 µL of perchloric acid solution. The sample solution was heated for 45 min at 60 °C. After cooling down to ambient temperature, the solution was detected at 548 nm after being diluted to 2.25 mL with glacial acetic acid. A calibration curve was established using ursolic acid as the reference compound. The amount of total triterpenoids was expressed as mg glycyrrhetinic acid equivalent per g of dried weight of hawthorn fruit (mg GLE/g DW). 

### 2.5. Evaluation of Antioxidant Activity

#### 2.5.1. DPPH Free Radical-Scavenging Activity Determination 

The stable DPPH was used to determine the free radical scavenging activity of the extracts [29]. A total of 0.1 mL of methanolic solution of DPPH (0.5 mM) was added into 0.1 mL of extract in various concentrations, and was left to stand for 30 min at room temperature. The absorbance of the reacted solution was detected at 517 nm. The experiment was repeated three times. The IC_50_ value denotes the concentration of the sample (μg/mL) that is required to scavenge 50% of the DPPH free radicals. 

#### 2.5.2. Trolox Equivalent Antioxidant Capacity (TEAC) 

A slightly modified method [30] for the TEAC (or the more specific definition as ABTS radical cation scavenging activity) evaluation was followed. The final concentrations of 0.1 mM ABTS^•+^, 4.4 unit/mL horseradish peroxidase (Sigma-Aldrich) and 50 μM H_2_O_2_ in 50 mM phosphate buffer (pH 7.4) were mixed well, and left to stand overnight in the dark at room temperature to form a blue-green color solution of stable ABTS^•+^ reagent. A total of 50 μL of sample solution was added into the ABTS^•+^ solution in a moderate proportion for measuring the decrease in absorbance at 734 nm after 10 min. The results were expressed as IC_50_ values, the concentration (μg/mL) of sample required for 50% inhibition of ABTS^•+^. 

### 2.6. α-Glucosidase Inhibitory Activity Assay

The inhibition activity of PEHE or WE on *α*-glucosidase was determined according to the method of Chu et al. [31], with slight modifications. PEHE sample (0–1 mg/mL) was added into aliquots of 20 µL of 100 mM phosphate buffer (pH 6.8) and 20 µL of 2.5 mM pNPG. After incubating at 37 °C for 5 min, 20 µL of α- Glucosidase (0.2 U/mL in 10 mM pH 6.8 phosphate buffer) was added and incubated at 37 °C for 15 min. The reaction was terminated by the addition of 80 μL of Na_2_CO_3_ (0.2M), and was determined spectrophotometrically at 405 nm using a microplate reader (Multiskan Spectrum, Thermo Electron Corporation, Waltham, MA, USA). The various concentrations of PEHE or WE were also used for the comparative study in 50% inhibition (IC_50_ values). All measurements were performed in triplicate.

### 2.7. Cell Experiment

#### 2.7.1. Cell Culture

A rat insulinoma cell line RIN-m5F (pancreatic β-cell, ATCC, CRL-11605) was purchased from the Bioresource Collection and Research Center (BCRC, No. 60410) of the Food Industry Research and Development Institute, Taiwan. RINm5F cells were maintained as a monolayer culture in complete medium at 37 °C in a humidified 5% CO_2_ incubator, with the medium changed every two days. The complete medium contained αMEM with folate (2 µM), thymidint (36 µM), hypoxanthine (36 µM), glycine (600 µM), serine (250 µM) and 10% fetal bovine serum. Penicillin (20 units/mL), streptomycin (0.02 mg/mL) and fungizone (2.5 μg/mL) were also added to the media to eliminate contamination.

#### 2.7.2. Cell Viability 

The previously described MTT method [32] was performed to evaluate the cell toxicity of fructose and extract. In brief, RIN-m5F cells at a density of 5 × 10^3^ cells/well were treated with different concentrations of fructose or extract as indicated. After 24 h of incubation, MTT (final concentration, 0.5 mg/mL) was added to each well and incubated for 2 h. The formed formazan crystal product was dissolved in DMSO, and the absorbance was read at 570 nm with an ELISA Reader (VersaMax, Molecular Devices, Sunnyvale, CA, USA).

#### 2.7.3. Intracellular ROS Level

RIN-m5F cells were seeded onto a 12-well plate at a density of 1 × 10^4^ cells/mL and incubated for 24 h. After treatment with fructose at a dose of 150 mM for 30 min, the PEHE was added at the indicated concentrations and incubated for an additional 4 h. The used culture medium was sucked off gently with a pipet, replaced with medium containing 10 µM DCFH-DA [33], and further incubated at 37 °C for 30 min. The medium was sucked off by using the pipet. The cultivated cells were then rinsed with cold PBS twice. Finally, the ROS levels were determined with an inverted fluorescence microscope (Olympus IX71, Tokyo, Japan). 

### 2.8. Gene Expression 

All of the following procedures for gene expression studies were achieved, as previously described [34].

#### 2.8.1. Extraction of RNA from RIN-m5F Cells

Cells at a density 4 × 10^5^ cells/mL were seeded onto a 6 cm dish and incubated for 24 h. The cells were pre-treated with fructose at 150 mM for 2 h, then treated with PEHE (50 and 100 μg/mL, respectively) or metformin (100 μM), and incubated for 24 h. The cultured medium was sucked off, and the cells were rinsed twice with ice-cooled PBS. A total of 1 mL of TRIzol^®^ Reagent (ThermoFisher Scientific, Waltham, MA, USA) was added and mixed for a while. Then, the culture was transferred into an Eppendorf vial, where 200 µL of chloroform was added and vortexed for 10 min, left to stand for 15 min and centrifuged at 13,000× *g* for 15 min. The supernatant (400 µL) was transferred into an Eppendorf vial; 600 µL of isopropanol was added, left to stand for 30 min, then centrifuged at 4 °C under 13,000 × *g* for 15 min, after which the supernatant was discarded. The sediment was rinsed with 500 µL of ethanol (70%), and centrifuged at 4 °C under 13,000 × *g* for 20 min. The washed sediment was re-dissolved in diethylpyrocarbonate water (Sigma-Aldrich) and stored at −80 °C for further use.

#### 2.8.2. Reverse Transcription of RNA to cDNA

The operation protocol was carried out by following instructions provided by the Takara PrimeScript^TM^ RT Reagent Kit (Takara Bio, San Jose, CA, USA). The final total volumes for each sample were all adjusted to 10 µL, incubated at 37 °C for 15 min, then reacted at 85 °C for 5 min and stored at −80 °C for use. A total of five genes involved in the ER stress and a reference gene of β-actin were evaluated in this study. Supplementary Materials Table S1 depicts the sequence of primers used in this study.

#### 2.8.3. Quantitative Analysis of mRNA Levels

An equal amount of cDNA was used for the subsequent qPCR performed with the SYBR^®^ FAST system (KAPA biosystems). The 20 μL reaction mixture contained 9.2 μL of cDNA, 0.4 μL of 10 uM forward and reverse primers, and 10 μL of KAPA SYBR FAST qPCR Master Mix (2×). Amplification was performed in the StepOnePlus™ Real-Time PCR System (Applied Biosystems). The DNA fragments were amplified for 40 cycles (enzyme activation: 20 sec at 95 °C hold; denaturation: 3 s at 95 °C; annealing: 40 s at 60 °C). The expression of β-actin was determined as the internal control. The relative expression level was calculated using the 2^−ΔΔ*C*t^ method.

### 2.9. Statistical Analysis

Means ± standard deviations (SDs) obtained from the studies were used to express the results. Statistical analysis of the results was determined using the GraphPad Prism program (5th edition, GraphPad, San Diego, CA, USA). One-way ANOVA was used for analysis of variations in each group, and Tukey’s post hoc test was applied for analysis of significance of differences among the means. A confidence level of *p* < 0.05 was considered to be statistically significant. 

## 3. Results

### 3.1. The Contents of Total Polyphenols, Flavonoids and Triterpenoids 

The TPC of the WE and PEHE were determined using the Folin–Ciocalteu method, with gallic acid as a standard. As shown in Table 1, PEHE (122.27 mg/g) had higher total polyphenol content than WE (56.53 mg/g). The TFC in the WE and PEHE were determined with the aluminum chloride (AlCl_3_) method, using quercetin as a standard. PEHE (192 mg/g) also showed a higher content of TFC than WE (24.98 mg/g) (Table 1). For the determination of TTC, a vanillin/glacial acetic acid solution was applied. Unexpectedly, the contents of total triterpenoid of WE (6.04 mg/g) and PEHE (6.61 mg/g) were fairly close (Table 1). It was supposed that the lower content of triterpenoids in the hawthorn fruit resulted may have resulted from indiscriminate and complete extraction by the water and organic solvent used, respectively. 

### 3.2. The Antioxidant Activities of WE and PEHE

**Figure 1:**
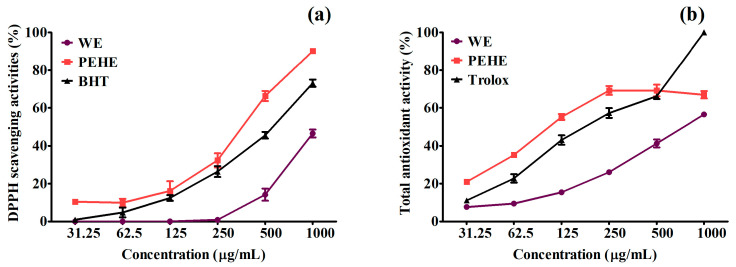
DPPH free radical (**a**) and ABTS radical cation (**b**) scavenging activities and IC_50_ values for scavenging free radicals from PEHE and WE of hawthorn fruits. Results are expressed as percentage decrease of absorbance at 517 nm (**a**) and 734 nm (**b**) with respect to blank. BHT and Trolox were used as standards, respectively. Data are expressed as mean ± SD (n = 3).

The free radical 2,2′-diphenyl-1-picrylhydrazyl (DPPH) scavenging activity and ABTS radical cation scavenging activity were widely applied to investigate the antioxidant activity of plant extracts. The disappearance of the DPPH radical at 515 nm detected spectrophotometrically implicates antioxidant activity of the prepared extracts; meanwhile, the ABTS assay was based on the generation of a blue/green ABTS^•+^ (detected at 734 nm) that can be reduced by antioxidant compounds. Figure 1a,b show the DPPH and the ABTS^•+^ radical-scavenging activities of WE, PEHE and the positive controls BHT and Trolox, respectively. PEHE exhibited significantly higher free radical scavenging abilities than WE. The results can be found significantly from the comparisons of IC_50_ values in Table 2, where the values of PEHE were significantly lower than WE for either DPPH or ABTS^•+^ radical scavenging activities. The PEHE (IC_50_ = 379.62 ± 23.21 µg/mL) exhibited stronger DPPH radical scavenging activity, and was even better than BHT (IC_50_ = 547.47 ± 9.69). On the other hand, the ABTS^•+^ radical scavenging activities also exhibited the same trend (Table 2).

These results demonstrated that liquid–liquid partition using ethyl acetate combined with the Sephadex^®^ LH–20 column separation on water extract of hawthorn fruit could effectively enrich polyphenol content in the extract, and lead to a further enhancement in antioxidant activity.

### 3.3. The Main Compounds in PEHE from HPLC–ESI–MS/MS Analysis

**Figure 2:**
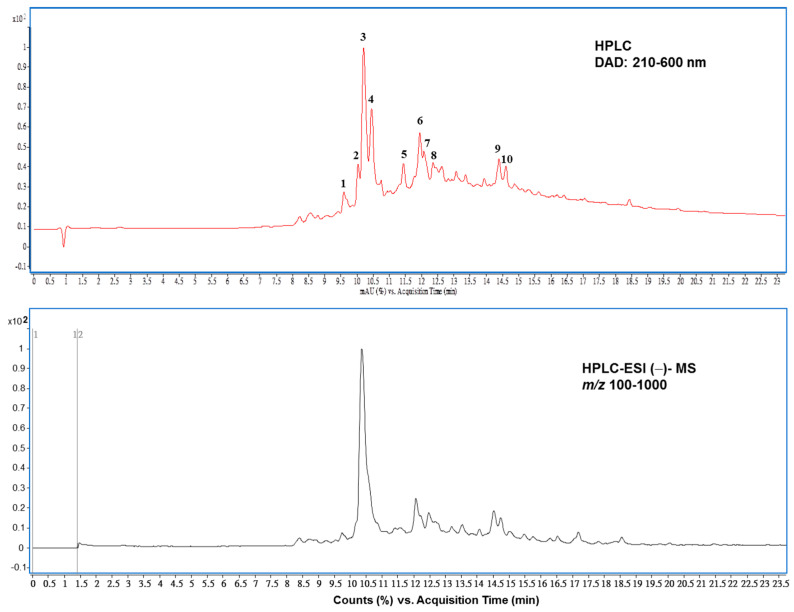
Representative total ion chromatogram of HPLC–MS spectrometry in the negative ionization mode (**bottom**), and HPLC (**top**) profile in the analyses of polyphenol-enriched hawthorn fruit extract (PEHE). Peak numbers refer to Table 3.

To investigate the compositions of the prepared extract enriched in polyphenols, two polarity modes in positive and negative ionizations of MS analyses were analyzed. As expected, the total ion chromatogram of the LC–MS measurement in negative ionization provided more significant characteristics of molecular ion than positive ionization mode (data not shown). This is in line with previously described observations that polyphenols are supposed to ionize in negative mode better than in positive mode with presentation of characteristic mass spectra [35]. As shown in Figure 2 (bottom panel), a reversed phase HPLC and mass spectrometry in negative ion mode method was developed, allowing for the separation and identification of ten components in PEHE analyzed with HPLC (Figure 1, top panel). 

As shown in Table 3, identification of the components existing in PEHE was carried out by comparing the retention times, UV-Vis spectra and mass spectra with those of authentic compounds when available. Results showed that one phenolic acid, two flavonoids and seven epicatechin derivatives were identified in the PEHE. The (epi)catechin derivatives showed characteristic UV spectra with λ_max_ 280 nm and fragment ions at *m*/*z* 285, which were also the characteristic basic skeleton of B-type procyanidins [36]. Epicatechin was found to be the highest, followed by epicatechin-4,8′-epicatechin-*C*-hexoside. These epicatechin derivatives occupied a total of more than 50% of the PEHE content. The second predominant group was found from flavonoid compounds, including quercein-3-*O*-galactoside (hyperoside) and quercertin-3-*O*-galactoside (isoquercitrin) (Table 3). The ESI (−)-MS/MS spectra of authentic compounds were identified, and are shown in Supplementary Materials Figure S1.

### 3.4. α-Glucosidase Inhibitory Activities of PEHE and WE

The α-glucosidase inhibitory activities can be used as a method to select antidiabetic phytochemicals for in vitro study through the hydrolysis inhibitory ability of α-glucosidase on *p*NPG. It was found that PEHE exhibited a lower IC_50_ value than WE (196.37 ± 23 vs. 2633.00 ± 215.32 µg/mL), indicating higher α-glucosidase inhibitory activity. Therefore, in view of the above-mentioned results of antioxidant activity and α-glucosidase inhibitory activity analyses, we choose PEHE for further cell experiments. 

### 3.5. Effects of Fructose and PEHE on Cell Viability 

**Figure 3:**
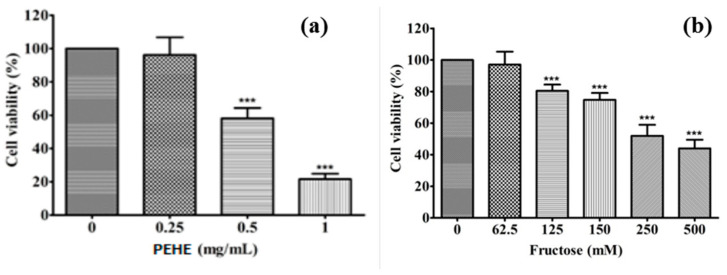
Effects of PEHE (**a**) and fructose (**b**) extract on RIN-m5F cell viability. RIN-m5F cells were treated with fructose or the polyphenol-enriched extract of hawthorn fruit (PEHE) for 24 h. Data are expressed as mean ± SD (n = 3); *** *p* < 0.001 compared to control.

In order to evaluate the effects of PEHE and fructose on cell proliferation, cells were treated with various concentrations of PEHE and fructose for 24 h, followed by the MTT assay. Cell viability was preserved with up to 0.25 mg/mL of PEHE treatment (Figure 3a); meanwhile, the effect of fructose in cell viability (<60%) was noticed after treatment with 250 mM of fructose (Figure 3b). These reductions were dose-responsive.

Considering the dose–effect relationship used in the expression of cell functions, the choices of concentrations of PEHE and fructose for the following experiments were determined at 50 and 100 μg/mL for PEHE (without any toxicity) and 250 mM for fructose (with acceptable cell viability of higher than 80% in inducing cell toxicity), respectively. 

### 3.6. PEHE Inhibits the Production of Reactive Oxygen Species Affected by Fructose

**Figure 4:**
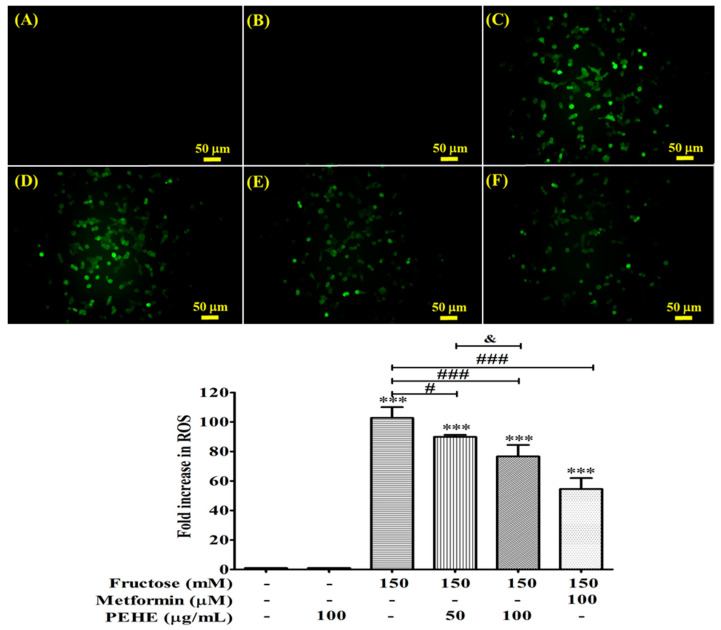
Intracellular ROS induced by fructose and attenuated by PEHE. RINm5F β-cells were pretreated with or without fructose (150 mM) for 2 h before exposure to PEHE (50 and 100 μg/mL) for 24 h. Intracellular reactive oxygen species were stained with DCFH-DA and assessed via phase contrast fluorescence microscopy [upper panel, original magnification (×100); (**A**) control, (**B**) PEHE (100 μg/mL) treatment only, (**C**) fructose (150 μg/mL) treatment only, (**D**) fructose + PEHE (50 μg/mL), (**E**) fructose + PEHE (100 μg/mL) and (**F**) fructose + metformin (100 μg/mL)]. One-way ANOVA was followed by Tukey’s post hoc test. Values are expressed as mean ± standard deviation (bottom panel, n = 3). *** *p* < 0.001 vs. the control group; # *p* < 0.05 and ### *p* < 0.001 vs. the fructose group. ^&^ *p* < 0.05.

To investigate whether oxidative stress alters β-cells functions, the cells were compared with those from fructose-treated cells. The invention of fructose (150 mM) significantly increased intracellular ROS levels (*p* < 0.001) up to 102.75 ± 7.39-fold compared to the control (Figure 4). PEHE reduced the increase in ROS by 11.31 ± 3.33% and 25.53 ± 2.70% at 50 and 100 μg/mL, respectively, compared to the fructose-induced ROS levels. Metformin reduced the ROS levels by 47.02 ± 3.65% at 100 μM (Figure 4). 

### 3.7. Effect of PEHE on Fructose-Induced ER Stress Signaling Pathway

**Figure 5:**
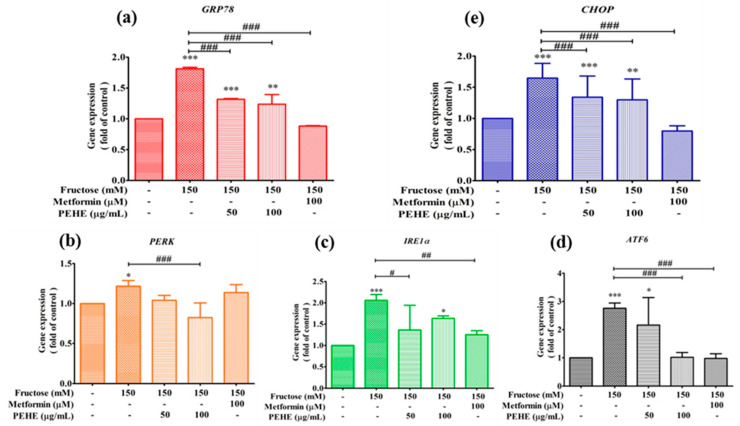
The effects of PEHE and metformin on fructose-induced ER stress relative gene expressions of GRP78 (**a**), PERK (**b**), IRE1α (**c**), ATF6 (**d**) and CHOP (**e**) in RINm5Fcell. Cells were pre-treated with 150 mM fructose for 2 h and then incubated, with or without PEHE for 24 h. The gene expressions were detected by using the StepOnePlus TM Real Time PCR System. One-way ANOVA was followed by Tukey’s post hoc test. Values are expressed as mean ± standard deviation (n = 3). * *p* < 0.05, ** *p* < 0.01 and *** *p* < 0.001 vs. the control group; ^#^ *p* < 0.05, ^##^ *p* < 0.01 and ^###^ *p* < 0.001 vs. the fructose group.

Exposure of RINm5F β-cells to a high concentration of fructose (150 mM) for 24 h induced a significant increase in the expression of ER stress-related genes (Figure 5a). Members of ER stress-related genes, including molecular chaperone GRP78 (an indicator of the onset of the unfolded protein response, UPR) and the key regulators of the UPR pathway PERK, IRE1α, ATF6 and CHOP, maintain an inactive state under homeostasis [20]. To evaluate the effect of high fructose treatment and the protection activity of PEHE on the changes of ER stress sensors, the aforementioned genes were detected. In the high fructose condition, the expression genes of GRP78 (Figure 5a) and the genes involved in UPR (i.e., PERK, IRE1α and ATF6, Figure 5b–d) and the apoptosis relative gene CHOP significantly increased in comparison with control cells, and the inhibitory effect on gene expression of PERK had a better effect than that of the anti-diabetic drug metformin. 

## 4. Discussion

A previous study using the water extract of hawthorn (*C. pinnatifida*) fruits to treat streptozotocin (STZ)-induced type II diabetes mellitus (T2DM) rats showed remarkable anti-diabetic effects [5]. However, in-depth studies of the functional components in the water extract of hawthorn fruit and its anti-diabetic mechanism have not been conducted. In this study, the composition of PEHE was analyzed with HPLC and LC–ESI–MS/MS, and its mechanism was illustrated by investigating the antioxidant activity, α-glucosidase inhibitory effect, cell protective effect, and inhibition capability on ER stress-related gene expression. 

The main phenolic compounds such as epicatechin and procyanidin B2, which belong to the flavan-3-ol monomers and dimers in the constituents of hawthorn fruit, have been indicated as the active compounds in reducing the oxidative stress [10,11]. In the purification and identification study, the isomer of epicatechin or catechin was finally confirmed from the ^13^C NMR spectrum [11]. Thus, the epicatechin relative compounds identified in the study (Table 3) were supposed to be the epicatechin derivatives, not the undecided (epi)catechin by the Karar et al. study [36]. As described above, these phenolic compounds extracted in the study may support the hypoglycemic effect observed in the in vivo study [5].

The major source of fructose is sucrose or table sugar, which is composed of one molecule of glucose and one molecule of fructose. The free fructose is absorbed after the ingestion and digestion of sucrose. The other major source of fructose is high-fructose corn syrup, with the composition of fructose and glucose mostly in the ratio of 45% glucose and 55% fructose. Excess ingestion of fructose has numerous effects on the brain, liver, vasculature, kidney, and adipocyte, resulting in metabolic syndrome and ultimately T2DM [37]. In the physiological response, an excess level of fructose has a deleterious effect on pancreatic β-cell function [37,38]. High fructose triggers oxidative modification and apoptosis in pancreatic β-cells; a study indicated that DNA cleavage, a change characteristic of apoptosis, is observed after 3 days of treatment with 100 mM fructose [39]. An increase in intracellular oxidative stress levels through high fructose treatment may provoke cell apoptosis. As mentioned above, pancreatic β-cells are sensitive to oxidative stress, owing to their lower intracellular antioxidant capacity [18,19]; excess oxidative stress may affect the survival of β-cells. Since β-cells’ functions gradually deteriorated under high fructose conditions, the present study is useful for understanding the protective effects of prepared high antioxidant PEHE (Figure 1 and Table 2) under hyperglycemic conditions.

In a study by Lin et al. [40], hyperoside, obtained through high-yield extraction from ethanol or acetone extraction on the same identified species of hawthorn fruits used in the study, has been reported to have strong antioxidant and anti-α-glucosidase properties. Moreover, through the results of molecular modeling docking, hyperoside also exhibited a high affinity with α-glucosidase. In addition to the various possible interactions between an inhibitor and enzyme, inhibition kinetics analysis is considered to be one of the main tools to distinguish the inhibition mechanisms involved [41]. As previously reported from the α-glucosidase inhibition study, the inhibition kinetics of polyphenols, which contain the main compounds of epicatechin and procyanidin B2, are a mixed-type of inhibitor with noncompetitive inhibition trends [41]. The significant antioxidant activity of polyphenols, as a crucial health-promoting factor, is one of the issues of concern. The extract PEHE also presented the highest DPPH free radical scavenging. In the study, the PEHE containing hyperoside (Table 3) also significantly showed DPPH free radical scavenging activity. In addition, an analysis of polyphenol antioxidants of hawthorn fruit found that the contents of total flavonoids, including free and bound, were between 5.589 and 6.667 mg GAE/g DW [42]. Flavonoids have been shown to exert a wide range of effects in biological systems, including potent radical scavenging activities [10,43], suggesting that these components also contribute to oxidative stress inhibitory properties. In addition, the antioxidant activity of hawthorn fruit has been demonstrated in a report by Lou et al., resulting from free phenolic compounds which are the main contributors (35.3–37.8%) to antioxidant activity in the fruit, with the most abundant being (-)-epicatechin, followed by procyanidin B2 [44].

A previous report described in detail the close relationships between oxidative stress and ER stress involved in β-cell dysfunction via direct effects on insulin biosynthesis and secretion [45]. Oxidative stress can cause ER stress and provoke multiple deleterious effects on β-cell function, including suppression of insulin transcription and the unfolded protein response (UPR) [45]. Under these conditions, ER stress activates the inactive heat-shock protein/chaperone, glucose-regulated protein GRP78 (also known as BiP), and initiates the activation and disassociation of three effectors, PKR-like ER kinase (PERK), inositol-requiring enzyme 1 (IRE1), and activating transcription factor 6 (ATF6) from chaperone GRP78, and follows by triggering the downstream UPR [45]. In the study, the gene expressions of the three ER stress sensors PERK, IRE1α and AFT6 were significantly activated after high-fructose introduction in beta-cells, while the PEHE treatment at 100 μg/mL ameliorated the damages (Figure 5b–d). Moreover, under non-homeostatic conditions, a driving apoptosis gene, C/EBP-homologous protein (CHOP), can be induced to an active status, and cause cell apoptosis. In Figure 5e, after treatment with fructose, the CHOP gene expression significantly increased 1.6-fold in comparison with the control. Meanwhile, the treatment of PEHE at 100 μg/mL significantly (*p* < 0.001) inhibited the gene expression. Therefore, since under hyperglycemic conditions CHOP plays an important role in the induction of β-cell apoptosis, the present study is critical for disclosing the therapeutic effects and understanding the active mechanisms of PEHE. The main constituents of PEHE, combined with epicatechin derivatives, procyanidins and flavonoids, presented significant inhibitory effects on free fatty acid-induced oxidative stress and expression of ER stress-related genes. The study of the relationship between antioxidant activity and the main constituents of PEHE suggest that procyanidin B2 acts as a more effective antioxidant agent than epicatechin, which in accordance with previous results [44,46,47]. However, it is difficult to speculate which compound(s) crucially affect the results of gene expression. This question remains to be resolved further through using purified compounds for conducting in-depth research.

## 5. Conclusions

We observed that the treatment of β-cells with a well-characterized PEHE strongly inhibited intracellular free radicals, and markedly influenced the expression of genes (i.e., GRP78, PERK, IRE1α, ATF6 and CHOP) related to ER stress. Using HPLC–ESI–MS/MS, the therapeutic activities of PEHE are possibly associated with the chemopreventive properties of polyphenols and flavonoids existing in the extract, especially the epicatechin derivatives. However, more research is required to confirm the therapeutic effect to which these findings may be applied to in vivo studies. 

## Figures and Tables

**Table 1:** Total polyphenols, flavonoids and triterpenoids of the water extract (WE) and the polyphenol-enriched extract (PEHE) prepared from ethyl acetate partition, followed by column fractionation on WE of hawthorn fruit.

Content (mg/g)	WE	PEHE
Total polyphenol (mg GAE/g dw) ^1^	56.53 ± 3.66 ^b^	122.27 ± 5.02 ^a^
Total flavonoid (mg QUE/g dw) ^2^	24.98 ± 1.09 ^b^	192.47 ± 13.96 ^a^
Total triterpenoid (mg GLE/g dw) ^3^	6.04 ± 0.12 ^b^	6.61 ± 0.02 ^a^

Each value represents the mean ± SD of triplicate experiments. Values with different letters within the same row are significantly different (*p* < 0.05). ^1,2,3^ The total phenol, flavonoid and triterpenoid contents were determined using the calibration curve equations of y = 1.2619x + 0.0055 (r^2^ = 0.9945), y = 0.3097x + 0.0466 (r^2^ = 0.998) and y = 0.0904x + 0.0104 (r^2^ = 0.9995), respectively.

**Table 2:** The DPPH and ABTS radical scavenging activities of WE and PEHE from hawthorn fruits.

IC_50_ (μg/mL)				
Free Radicals	WE	PEHE	BHT	Trolox
DPPH	1057.36 ± 41.98 ^a^	379.62 ± 23.21 ^c^	547.47 ± 9.69 ^b^	--
ABTS^•+^	784.93 ± 19.11 ^a^	108.99 ± 3.05 ^c^	--	186.04 ± 22.60 ^b^

Each value represents the mean ± SD of triplicate experiments. Values with different letters within the same row are significantly different (*p* < 0.05).

**Table 3:** HPLC–DAD–ESI–MS/MS analysis on the chromatographic and spectroscopic characteristics and individual content identified in the polyphenol-enriched extract (PEHE) of hawthorn fruit.

Peak No. ^a^	RT (min)	Compound Name	λ_max_ (nm)	[M−H]^−1^ *m/z*	MS/MS ^d^ *m/z*	Area % ^e^
1	9.58	Epicatechin- 4,8′-epicatechin *C*-hexoside ^c^	280	739 ^c^	289, 449, 329, 467	3.86
2	10.03	Procyanidin B2 ^b^	282	577	407, 289, 125, 245	5.25
3	10.20	Epicatechin ^b^	230, 278	289	109, 245, 203, 123	35.64
4	10.44	Epicatechin-4,8′-epi catechin *C*-hexoside ^c^	224, 280	739	289, 197, 449, 167	18.91
5	11.43	Sinapic acid *O*-hexoside ^c^	280	385	223, 179	3.06
6	11.94	Epicatechin derivative	280	401	289, 245, 203, 109	6.19
7	12.06	Procyanidin C1 ^c^	280	865	287, 695, 245, 407	2.99
8	12.35	Epicatechin derivative	280	647	289, 221, 245, 137	1.18
9	14.39	Quercein-3-*O*-galactoside ^b^	270, 352	463	300, 301, 271, 255	3.88
10	14.60	Quercetin-3-*O*-glucoside ^b^	270, 352	463	300, 301, 271, 255	2.11
						83.07

^a^ Peaks numbers refer to Figure 2. ^b^ Compound identification by comparison with authentic standards. ^c^ Compound identification by comparison with the literature [11,36]. ^d^ MS/MS fragment ions are shown in decreasing order according to their signal intensity. ^e^ The calculations are based on considering the HPLC analysis detected at wavelengths of 210–600 nm on compounds existing in PEHE as 100%.

## Data Availability

Data are contained within the article or the Supplementary Materials.

## Supplementary Materials

The following supporting information can be downloaded at: https://www.mdpi.com/article/10.3390/foods12061130/s1, Figure S1: The ESI (−)-MS/MS spectra of the four authentic standards. Numbers are referred to Table 3. Table S1: Primers for real-time PCR analyses.

## Abbreviations

ABTS	2:2′-azinobis-(3-ethylbenzothiazoline-6-sulfonate)
ATF6	activating transcription factor–6
CHOP	CCAAT-enhancer-binding protein (C/EBP) homologous protein
DCFH-DA	dichloro-dihydro-fluorescein diacetate
ER	endoplasmic reticulum
GRP78	glucose-regulating protein 78
IC_50_	half maximal inhibitory concentration
IRE1α	inositol-requiring enzyme 1α
PERK	protein kinase R-like endoplasmic reticulum kinase
UPR	unfolding protein response
