# Supplementary material for: Protective Effect of Hawthorn Fruit Extract against High Fructose-Induced Oxidative Stress and Endoplasmic Reticulum Stress in Pancreatic β-Cells
Source: Foods. 2023 Mar 8;12(6):1130. doi: 10.3390/foods12061130 (PMC10047983; doi:10.3390/foods12061130)
Supplement: Supplementary file 1 [file foods-12-01130-s001.zip › foods-2188282-supplementary.pdf]

# Supplementary Table S1

# Protective Effect of Hawthorn Fruits Extract against High Fructose-Induced Oxidative Stress and Endoplasmic Reticulum Stress in Pancreatic β-cells

**Hsiu-Man Lien[1,2,*], Hsin-Tang Lin[3], Shiau-Huei Huang[1],Yìng-Ru Chen[1], Chao-Lu Huang[2], Chia-Chang Chen[2] and Charng-Cherng Chyau[1,*]**

[1] Research Institute of Biotechnology, Hungkuang University, Shalu District, Taichung 43302, Taiwan; ccchyau@hk.edu.tw (C.-C.C.); a7651618@yahoo.com.tw (S.-H.H.); wk00552@gmail.com (Y.-R.C.)
[2] SYi Biotek, 2F, No. 26, Keyuan Rd., Xitun District, Taichung 40763, Taiwan; lien736@gamil.com (H.-M.L.); chaoluhng@gmail.com (C.-L.H.); casey5115@gmail.com (C.-C.C.)
[3] Graduate Institute of Food Safety, National Chung Hsing University, 145, Xingda Road, Taichung City 40227, Taiwan; linhs@nchu.edu.tw (H.-T.L.)
*Correspondence: lien736@gamil.com (H.-M.L.); ccchyau@hk.edu.tw (C.-C.C.)

**Table S1.** Primers for real-time PCR analyses.

| Name (Accession No.) | Sequence (5′ to 3′) | Product Length (bp) |
|---|---|---|
| *GRP78* (NM_013083.2) | F: AGAAACTCCGGCGTGAGGTAGA<br>R: TTTCTGGACAGGTTTCATGGTAG | 176 |
| *ATF6* (NM_001107196.1) | F: GGGAGTGAGCTGCAGGTGTA<br>R: TTATGGGTGGTAGCTGGTAA | 137 |
| *PERK* (NM_019356.1) | F: GCTTGCTATGGTTACGAAGGC<br>R: CATCACATACCTGGGTGGAG | 120 |
| *IRE1α* (NM_001191926.1) | F: CGAGCCATGAGGAATAAGAG<br>R: GGAAACGTGATGTGAAGTAG | 109 |
| *CHOP(GADD153)* (NM_024134.2) | F: AGCTGGAAGCCTGGTATGAGGA<br>R: AGCTAGGGATGCAGGGTCAA | 134 |
| *actin* (NM_007392) | F: TCTCCACCTTCCAGCAGATGT<br>R:AGCTCAGTAACAGTCCGCCTAGA | 100 |

F: Forward, R: Reverse

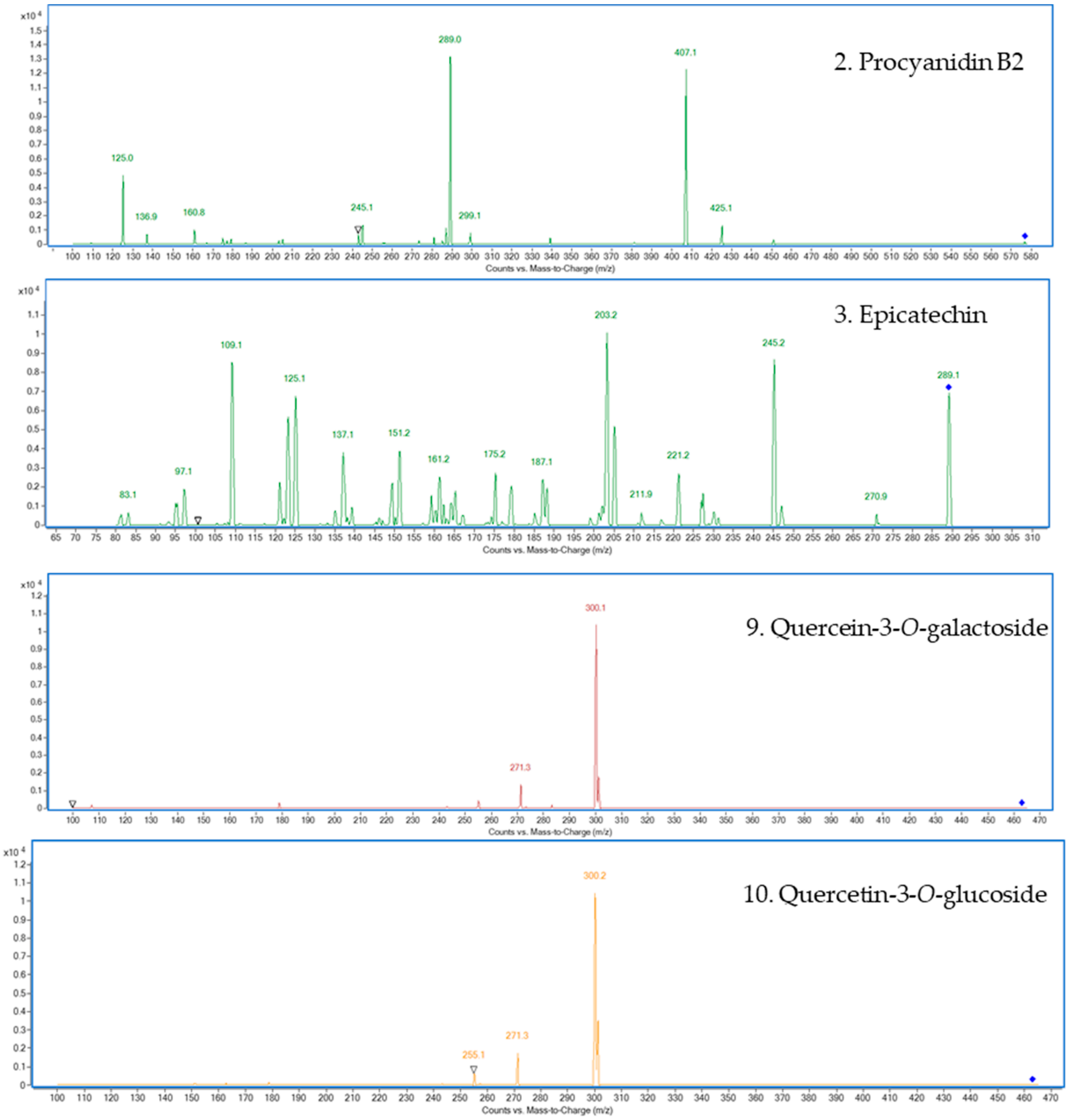


Figure S1. The ESI(−)-MS/MS spectra of the four authentic standards. Numbers are referred to Table 3.
